# Hypothalamic Expression of *KiSS1* and RFamide-related Peptide-3 mRNAs during The Estrous Cycle of Rats

**Published:** 2013-03-03

**Authors:** Mohammad Saied Salehi, Mohammad Reza Jafarzadeh Shirazi, Mohammad Javad Zamiri, Farid Pazhoohi, Mohammad Reza Namavar, Ali Niazi, Amin Ramezani, Nader Tanideh, Amin Tamadon, Afsoon Zarei

**Affiliations:** 1 Department of Animal Science, College of Agriculture, Shiraz University, Shiraz, Iran; 2 Histomorphometry and Stereology Research Center , Department of Anatomical Sciences, School of Medicine, Shiraz University of Medical Sciences, Shiraz, Iran; 3 Biotechnology Institute, Shiraz University, Shiraz, Iran; 4 Department of Medical Biotechnology, School of Advanced Medical Sciences and Technologies, Shiraz University of Medical Sciences, Shiraz, Iran; 5 Stem Cell and Transgenic Technology Research Center and Department of Pharmacology, School of Medicine, Shiraz University of Medical Sciences, Shiraz, Iran; 6 Department of Clinical Sciences, School of Veterinary Medicine, Shiraz University and Infertility Research Center, Shiraz University of Medical Sciences, Shiraz, Iran; 7 Infertility Research Center & IVF Center, Shiraz University of Medical Sciences, Shiraz, Iran

**Keywords:** Hypothalamus, *KiSS1*, RFamide-related peptide-3, Estrous Cycle, Rat

## Abstract

Kisspeptin and *RFamide-related peptide-3* (*RFRP-3*) are known to affect GnRH/luteinizing hormone (LH) in several species, including the rat. It has been hypothesized that GnRH/LH changes during the rat estrous cycle may result from changes in the expression of *KiSS1* and *RFRP-3* genes. Therefore, the present study investigates *KiSS1* and *RFRP-3* gene expression at the transcriptional level in the rat hypothalamus during the estrous cycle.

In the present experimental study, 36 adult female Sprague-Dawley rats (3-4 months old) were used to study the expression of *KiSS1* and *RFRP-3* mRNA in the hypothalamus during the estrous cycle. Four rats were ovariectomized, whereas the remainder were allotted to four different phases of the estrous cycle (n=8 per estrus phase). Rats were decapitated, and the hypothalami were immediately dissected and frozen in liquid nitrogen. Expressions of *KiSS1* and *RFRP-3* mRNAs were analyzed by real-time PCR.

The expression of *KiSS1* mRNA during estrus was lower than other phases of the cycle (p<0.01). Expression of *KiSS1* mRNA during the metestrus phase was lower than the proestrus phase (p<0.01). The expression of *RFRP-3* mRNA during proestrus was lower than the diestrus phase (p<0.01).

Results of the present study showed the role of coordinated expression of *KiSS1* and *RFRP-3* mRNA in the hypothalamus in the control of the rat estrous cycle.

Kisspeptins belong to a family of peptides encoded by the *KiSS1* gene and are natural ligands of the GPR54 receptor. Kisspeptin has a fundamental role in control of the gonadal axis (1, 2) . It has been shown that kisspeptin neurons are located upstream of GnRH neurons, and by affecting these cells, stimulate luteinizing hormone (LH) release (3). Because the excitatory effect of kisspeptin on gonadotropin secretion is inhibited by GnRH antagonists (4) and as kisspeptin administration to hypothalamo-pituitary disconnected ewe models could not change LH concentration (5), it has been concluded that kisspeptin acted at the hypothalamic level, not the pituitary, to stimulate GnRH release. Most GnRH neurons express GPR54 mRNA (6) and many kisspeptin neurons in rats express ERα (7). Thus it is possible that estrogen effects on GnRH cells are mediated through these neurons.

The *RFamide-related peptide-3* (*RFRP-3*) that putatively modulates the negative feedback effect of estrogen on gonadotropin secretion has been identified in the brain of rodents. The RFRPir cells, clustering in the dorsomedial nucleus of the hypothalamus (DMH), have been identified in hamsters, rats, and mice (8). Inhibitory effects of *RFRP-3* on gonadotropin release were reported in rodents (9, 10) and sheep (11, 12).

The pattern of LH secretion during the estrous cycle is a reflection of GnRH release. In the rat, serum levels of LH are lowest in early estrus, shortly after ovulation, and through metestrus, diestrus, and midproestrus. On the afternoon of proestrus, the circulating levels of LH begin to rise rapidly and peak in the evening, resulting in ovulation. The blood LH level then decreases and reaches basal levels by the early morning of the estrus phase (13) . During the estrus, metestrus, diestrus, and early proestrus, the concentration of GnRH is at its basal level. At mid-proestrus, the GnRH surge center is activated, which increases the GnRH concentration (14). With regards to the changing patterns of GnRH/LHs during the estrous cycle and due to the excitatory effect of kisspeptin and inhibition of *RFRP-3* on GnRH/ LH, we have hypothesized that GnRH/LH changes might result from changes in the expression of *KiSS1* and *RFRP-3* genes. Simultaneous evaluation of *KiSS1* and *RFRP-3* mRNA expressions in the rat hypothalamus has not been studied. The aim of the present study was to investigate *KiSS1* and *RFRP-3* gene expression at the transcriptional level in the hypothalamus during the estrous cycle of the rat.

A total of 36 adult (3-4 months old) female Sprague-Dawley rats (Rattus norvegicus) that weighed 170-220 g were used in this study. The rats were randomly selected and housed in the Laboratory Animal Center of Shiraz University of Medical Sciences, Shiraz, Iran under controlled temperature (22°C) and light (12:12 light to dark ratio; lights on at 7:30 AM) conditions. Rats were treated humanely and in compliance with the recommendations of the Animal Care Committee of the Shiraz University of Medical Sciences. All experimental procedures were carried out between 12.00-2.00 pm. Vaginal smears were prepared as previously described (15) for identification of the phases of the estrous cycles of the 32 intact rats. We assigned 8 rats to each phase of the cycle.

The control group comprised 4 randomly selected ovariectomized rats. The rats were anesthetized by an intraperitoneal injection of ketamine (100 mg/kg, Woerden, Netherlands) and xylazine (7 mg/kg, Alfazyne, Woerden, Netherlands), then ovariectomized through a ventral midline incision. Further procedures were undertaken after a twoweek recovery period.

The cyclic and ovariectomized rats were decapitated, brains dissected out immediately, and the entire hypothalami were dissected by the following procedure: an anterior coronal section, approximately 1 mm anterior to the optic chiasma, and a posterior coronal cut at the posterior border of the mammillary bodies were made (16, 17). A small portion of the thalamus located above the hypothalamus was dissected out. There is no kisspeptin expression in the thalamus (18). No other tissues such as the subthalamus, epithalamus or any other parts of the brain were dissected along with the hypothalamus, because the posterior cut was located at the posterior border of the mammillary bodies. The sub-samples were frozen in liquid nitrogen and stored at -80°C. For extraction of mRNA, at least 100 mg of tissue was needed. We were unable to extract mRNA from separate areas of the hypothalamus. Thus, instead of using a single nucleolus from the hypothalamus to evaluate relative expression of *RFRP-3*/*KiSS1* mRNA, all hypothalami were used which was consistent with studies of Navarro et al. (19) and Roa et al. (20).

Total RNA was extracted with RNX-Plus buffer (Cinnagen, Iran). Briefly, approximately 100 mg of the tissue were ground in liquid nitrogen. The ground powder was transferred to 1 ml of RNXplus buffer in an RNase-free microtube, mixed thoroughly and maintained at room temperature for 5 minutes. Chloroform (0.2 ml) was added to the slurry and mixed gently. The mixture was centrifuged at 15500 g at a temperature of 4°C for 15 minutes; the supernatant was transferred to another tube and precipitated with an equal volume of isopropanol for 15 minutes on ice. The RNA pellet was washed with 75% ethanol, quickly dried and resuspended in 50 µl of RNase-free water. The purified total RNA was quantified by a Nano-Drop ND 1000 spectrophotometer (USA). The DNase treatment was performed with a DNase kit (Fermentas, Germany) according to the manufacturer’s instructions. The DNase-treated RNA (3 µg) was used for first strand cDNA synthesis, by using 100 pmol oligo-dT (18 mer), 15 pmol dNTPs, 20 U RNase inhibitor and 200 U M-Mulv reverse transcriptase (all from Fermentas, Germany) in a 20 µl final volume. We designed the primer with Allele ID 7 software for the reference gene, *KiSS1* (NM_181692) and *RFRP-3* (NM_023952). The rat glyceraldehyde-3-phosphate dehydrogenase (GAPDH) gene (M32599) was used as reference gene for data normalization (Table 1).

**Table 1:** Sequences of real-time PCR primers used to evaluate relative expression of RFamide-related peptide-3 (RFRP-3) and *KiSS1* genes in the rat


Primer	Sequence	Amplicon length (bp)

**KiSS1-F**	TGCTGCTTCTCCTCTGTG	116
**KiSS1-R**	CCAGGCATTAACGAGTTCC	
**RFRP-3-F**	CTCAGCAGCCAACCTTCC	165
**RFRP-3-R**	AAACCAGCCAGTGTCTTG	
**GAPDH-F**	AAGAAGGTGGTGAAGCAGGCATC	112
**GAPDH-R**	CGAAGGTGGAAGAGTGGGAGTTG	


GAPDH;Glyceraldehyde-3-phosphate dehydrogenase.

A 30 µl volume of purified PCR product (for *KiSS1*, *RFRP-3* and GAPDH) was sent to Tech Dragon Limited Company for sequencing. We performed similarity search and sequence analysis using the BLAST server of the NCBI gene bank. Relative real-time PCR was performed in a 20 µl volume that contained 1 µl cDNA, 1x Syber Green buffer and 4 pmol of the primer. The amplification reactions were carried out in a Line-Gene K thermal cycler (Bioer, China) under the following conditions: 2 minutes at 94°C, 40 cycles at 94°C for 10 seconds, 57°C for 15 seconds and 72°C for 30 seconds. After 40 cycles, the specificity of the amplifications was tested by heating from 50°C to 95°C, which resulted in melting curves. We repeated all amplification reactions three times under identical conditions, including a negative control and five standard samples. To ensure the PCR products were generated from cDNA and not genomic DNA, proper control reactions were carried out in the absence of reverse transcriptase. For quantitative real-time PCR data, we calculated relative expression of *KiSS1* and *RFRP-3* based on the threshold cycle (CT) method. C_T_ for each sample was calculated using Line-gene K software (21). Fold expression of the target mRNAs over reference values was calculated by the equation 2-ΔΔC_T_ (22), where ΔC_T_ is determined by subtracting the corresponding GAPDH C_T_ value (internal control) from the specific C_T_ of the target (*KiSS1* or *RFRP-3*). ΔΔC_T_ was obtained by subtracting the ΔC_T_ of each experimental sample from that of the calibrator sample (ovariectomized rats).

Data on the relative expression of *KiSS1* and *RFRP-3* genes, and the ratio of *RFRP-3*/*KiSS1* were subjected to the test of normality. Analysis of variance for both variables were performed using Proc GLM (SAS, 2002) followed by mean comparison by Duncan`s multiple range test. We considered p<0.01 as significant. Group means and their standard error have been reported in the text.

Expression of *KiSS1* mRNA in the hypothalamus of female rats at different phases of the estrous cycle is shown in figure 1.

**Fig 1:**
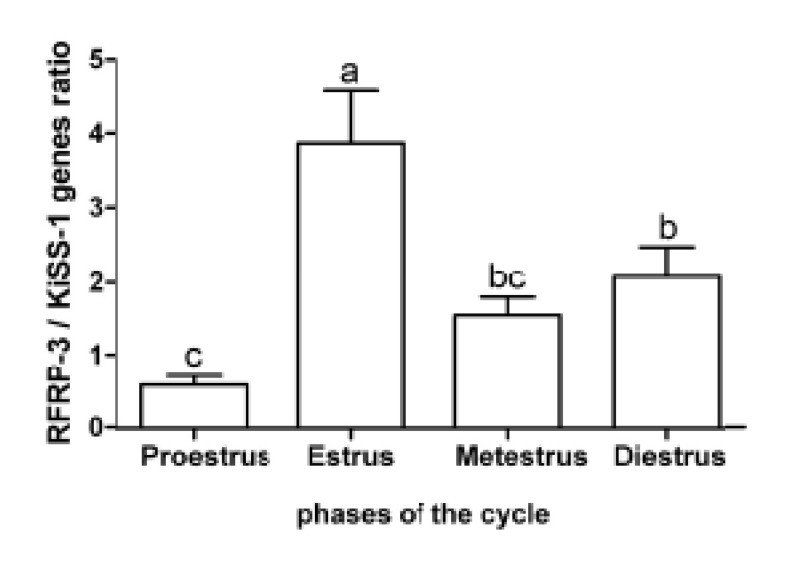
Mean (± standard error) of the relative expression of KiSS1 gene in the hypothalamus of rats (n=8) during the estrous cycle. Different letters indicate significant difference (p<0.01).

There was lower expression of *KiSS1* mRNA during the estrus phase (28.61 ± 3.48) compared to the other phases of the cycle (p<0.01). Expression of *KiSS1* mRNA during metestrus (62.78 ± 7.98) was lower than proestrus (94.62 ± 10.57, p<0.01). The expression of *KiSS1* mRNA during the diestrus phase did not significantly differ from the metestrus and proestrus phases.

Expression of *RFRP-3* mRNA during proestrus (49.06 ± 7.92) was lower than the diestrus phase (126.41±17.89, p<0.01, Fig 2).

**Fig 2:**
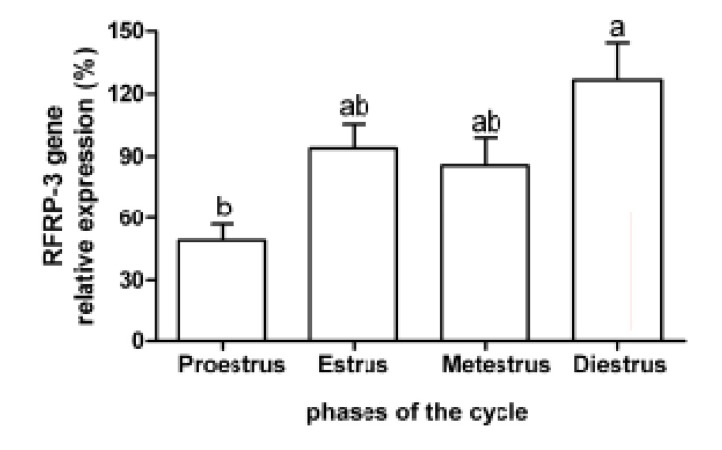
Mean (± standard error) of the relative expression of RFamide-related peptide-3 (RFRP-3) gene in the hypothalamus of rats (n=8) during the estrous cycle. Different letters indicate significant difference (p<0.01).

Intermediate values were found during the estrus (94.07 ± 11.43) and metestrus (85.96 ± 13.04) phases, which did not significantly differ from the observed values during the proestrus and diestrus phases. The expression ratio of *RFRP-3* and *KiSS1* mRNA in the proestrus and estrus phases were obviously contrariwise. There was a higher expression ratio of *RFRP-3*:*KiSS1* mRNA during the estrus (3.85 ± 0.75) phase compared to the other phases of the estrous cycle (p<0.05, Fig 3).

**Fig 3:**
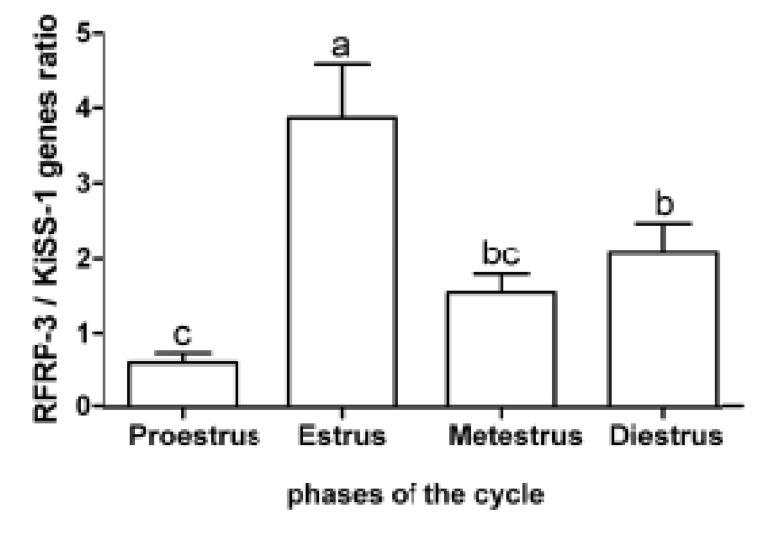
Mean (± standard error) of the relative expression of RFamide-related peptide-3 (RFRP-3) gene in the hypothalamus of rats (n=8) during the estrous cycle. Different letters indicate significant difference (p<0.01).

This ratio in the diestrus (2.04 ± 0.42) phase was more than proestrus (0.59 ± 0.13, p<0.05). The expression ratio of *RFRP-3*:*KiSS1* mRNA during estrus (1.51 ± 0.25) did not significantly differ with the diestrus and proestrus phases. The ratio of *RFRP-3*:*KiSS1* expressions were: proestrus (1:2), estrus (4:1) and diestrus (2:1).

The lowest expression of *KiSS1* mRNA in the hypothalamus was observed during the estrus phase. Studies using immunohistochemistry and in situ hybridization showed that kisspeptin peptide and *KiSS1* mRNA were concentrated in the arcuate nucleus (ARC) and anteroventral periventricular nucleus (AVPV) of rodents (23, 24). A small population of kisspeptin neurons were also identified in the preoptic periventricular nucleus (PeN) (23). Ovariectomy led to a significant increase in *KiSS1* mRNA in the entire hypothalamus of rats while this increase in gene expression was not observed in estradiol-implanted ovariectomized animals (19). Another study, using in situ hybridization, reported an increase in *KiSS1* mRNA of the mouse hypothalamus as a result of increases in *KiSS1* mRNA expression in kisspeptin neurons in the ARC. It was suggested that kisspeptin neurons of the ARC probably mediated the negative feedback effect of estrogen on gonadotropin secretion, which might be the GnRH pulse generating center. In contrast to ARC, there was reduced *KiSS1* mRNA expression in AVPV and PeN after ovariectomy, which increased after estradiol implantation. It was hypothesized that AVPV might have a role in preovulatory GnRH/LH surge (25). Another study reported that although kisspeptin neuronal activity in AVPV increased with estrogen treatment and could have a role in the preovulatory surge, kisspeptin neuronal activity in ARC was inhibited by estrogen which could act as a site for the negative feedback of steroids on GnRH and LH secretion (7). Investigation of *KiSS1* mRNA expression in ARC and AVPV at different phases of the estrous cycle in rats has shown the highest *KiSS1* mRNA levels in AVPV during proestrus and lowest during metestrus. This is consistent with the hypothesis that kisspeptin neurons in AVPV are regulated by the estrogen positive feedback. The level of *KiSS1* mRNA in ARC has been shown to be highest during diestrus and lowest during proestrus. It seems that during the estrous cycle, kisspeptin expression in ARC gradually increases from proestrus to the diestrus phase (26).

Results of the present study showed that *KiSS1* mRNA expression in the hypothalamus was higher during proestrus than estrus. Consistent with these findings, Adachi et al. (26) have determined that gene expression during proestrus is highest in AVPV and lowest in ARC. The *KiSS1* mRNA expression in ARC is negatively regulated by estrogen (27). The lowest level of *KiSS1* mRNA expression has been recorded during estrus, which agreed with the low level of gene expression in these nuclei at this stage of the cycle. High levels of *KiSS1* mRNA were also recorded during metestrus and diestrus, a finding consistent with the high level of ARC gene expression during the cycle (26).

The relative expression of *RFRP-3* mRNA was higher during diestrus compared with the proestrus phase. Consistent with these results, the number of RFRP-expressing neurons and the percentage of FOS-expressing RFRP neurons in hamsters were higher during diestrus than in the evening of proestrus. *RFRP-3* peptide cells only exist in the DMH of rodents (8). *RFRP-3* has an inhibitory effect on the GnRH neuronal system in rats (28) and mice (29). Approximately 40% of RFRP neurons in the DMH of hamsters (8) and about 18% of these neurons in ovariectomized mice (29) express ERα and the axon terminals of these neurons project to the GnRH neurons in mice, rats and hamsters (8). *RFRP-3* cause hyperpolarization (30) and decrease the electrical activity of GnRH neurons (31); administration of 17β-estradiol significantly reduced prepro-RFRP mRNA expression in ovariectomized mice (29).

In rats, ovarian estradiol secretion during the estrus phase is low, while at the end of metestrus estrogen secretion begins to increase and is high during the diestrus phase; it peaks in the proestrus evening, thereafter declining to its basal level (13). As administration of *RFRP-3* inhibits GnRH neuronal activity during an estrogen-induced LH surge (32) it seems that *RFRP-3* influence is absent at this period. Maximal estradiol concentration in the estrous cycle of rats occurs during proestrus, whereas minimal levels are seen during estrus. Therefore, in support of our findings, it seems that the high estradiol concentration secreted in the evening of the proestrus phase from dominant ovarian follicles stimulates kisspeptin neurons of the AVPV and inhibits RFRP neurons in DMH.

A decrease in the *RFRP-3*:*KiSS1* ratio guarantees the possibility of surge occurrence. Therefore, a high concentration of estradiol during the proestrus phase leads into GnRH/LH surge and ovulation. On the other hand, estradiol secretion in the estrus phase is at basal level. Therefore, removing its inhibitory effect on RFRP neurons increases the *RFRP-3*:*KiSS1* ratio. Medium concentrations of this steroid are secreted in the afternoon of diestrus (13). Therefore, it is probable that increase in RFRP expression along with secretion of progesterone from the corpus luteum inhibits the GnRH/LH surge and subsequent ovulation during diestrus.

The ratio of *RFRP-3*:*KiSS-1* expression at proestrus, estrus and diestrus was 1:2, 4:1, and 2:1, respectively. Results of the present study showed the role of coordinated expression of *KiSS1* and *RFRP-3* mRNA in the hypothalamus in the control of the rat estrous cycle.
